# Nonstructural proteins 2C and 3D are involved in autophagy as induced by the encephalomyocarditis virus

**DOI:** 10.1186/1743-422X-11-156

**Published:** 2014-09-01

**Authors:** Lei Hou, Xinna Ge, Lingxiang Xin, Lei Zhou, Xin Guo, Hanchun Yang

**Affiliations:** Key Laboratory of Animal Epidemiology and Zoonosis of the Ministry of Agriculture, College of Veterinary Medicine and State Key Laboratory of Agrobiotechnology, China Agricultural University, No. 2 Yuanmingyuan West Road, Haidian District, Beijing, 100193 People’s Republic of China

**Keywords:** Encephalomyocarditis virus (EMCV), Nonstructural protein, 2C, 3D, Autophagy, Endoplasmic reticulum (ER) stress, Unfolded protein response (UPR)

## Abstract

**Background:**

Encephalomyocarditis virus (EMCV) can infect a variety of animal species and humans. Although the EMCV infection is known to induce autophagy to promote its replication in host cells, the viral proteins that are responsible for inducing autophagy are unknown.

**Methods:**

The recombinant plasmids that were expressing the EMCV proteins were constructed to analyze the role of each protein in the induction of autophagy. Autophagy inductions by the EMCV proteins in BHK-21 cells were investigated by confocal microscopy, Western blotting and transmission electron microscopy. ER stress in BHK-21 cells was examined by detecting the marker molecules using western blotting and luciferase assays.

**Results:**

This study presents the first demonstration that the nonstructural proteins 2C or 3D of EMCV were involved in inducing autophagy in BHK-21 cells that were expressing 2C or 3D, and we found that inhibiting Beclin1 expression influenced this autophagy induction process. Next, 2C and 3D were shown to be involved in inducing autophagy by activating the ER stress pathway. Finally, EMCV 2C or 3D were demonstrated to regulate the proteins associated with PERK and ATF6alpha pathway.

**Conclusions:**

Our findings indicate that 2C and 3D are involved in EMCV-induced autophagy by activating ER stress molecules and regulating the proteins expression associated with UPR pathway, helping to better understand the EMCV-induced autophagy process.

**Electronic supplementary material:**

The online version of this article (doi:10.1186/1743-422X-11-156) contains supplementary material, which is available to authorized users.

## Background

Encephalomyocarditis virus (EMCV) belongs to the *Cardiovirus* genus of the *Picornaviridae* family [1]. This virus has a wide host-range among domestic and wild animals [2–4]. Out of all the domestic animals, pigs are considered the most commonly and severely EMCV-infected animals [5]. EMCV is not only an important pathogen in animal husbandry, but it also has potential public health significance [6]. Therefore, understanding the specific interaction between EMCV and hosts/cells is required for the effective treatment and control of this infection.

EMCV is a nonenveloped, single-stranded, positive-sense RNA virus. The genome is approximately 7.8-kb long with a single open reading frame (ORF) that is translated into a polyprotein precursor [7]. This precursor is proteolytically processed into structural proteins (VP1, VP2, VP3 and VP4), primarily forming the viral nucleocapsid and nonstructural proteins (2A, 2B, 2C, 3A, 3B, 3C and 3D) along with several protein intermediates that are needed for viral replication [8]. Although the roles of EMCV proteins have been widely investigated, a further exploration of the virus-host interaction and important cellular components in the EMCV life cycle is essential to determine a control strategy for EMCV infection.

The endoplasmic reticulum is one origin of the membranes that generate autophagosomes [9, 10]. The endoplasmic reticulum is also a multifunctional organelle in eukaryotic cells, which provides a unique compartment for posttranslational modifications, folding, and the oligomerization of newly synthesized membrane and secreted proteins. However, several endogenous imbalances in cells often contribute to ER malfunction known as ER stress [11]. In response to ER stress, a coordinated adaptive program called the unfolded protein response (UPR) is activated and serves to minimize the accumulation and aggregation of misfolded or over-expressed proteins by increasing the capacity of the ER machinery to fold correctly and degrade aberrant proteins. To date, three ER stress sensors, namely IRE1 (inositol-requiring enzyme 1), ATF6α (activating transcription factor 6α), and PERK (PKR-like ER protein kinase), have been identified in mammals for their ability to achieve different cellular adaptations [12].

Autophagy is a dynamic, conserved intracellular process that involves the formation of a characteristic double- or single-membrane structure (autophagosomes and autolysosomes, respectively), which delivers misfolded or long-lived cytoplasmic proteins and damaged or obsolete organelles to lysosomes for digestion and recycling [10, 13–16]. Autophagy not only plays an important role in cellular homeostasis, but it also acts as a cellular response to stress such as pathogen infection [17–19]. Some RNA viruses may subvert the defensive function of autophagy and use the autophagic double- or single-membrane vesicles to facilitate their own replication [20–22].

In recent years, a lot of attention has been paid to the relation between autophagy and viral infection [23, 24]. Our previous study indicated that EMCV infection can induce autophagy in host cells, and it is able to facilitate viral replication [25]. Thus, to address which protein(s) in EMCV is (are) involved in autophagy induction is necessary to understand the interaction between autophagy and viral infection. In this study, we firstly demonstrated that autophagy is induced in EMCV 2C or 3D protein-expressing cells by monitoring the presence of autophagosome-like vesicles and modifying LC3, the mammalian Atg8 known as homolog microtubule-associated protein light chain 3. Moreover, we observed the interference effect of Beclin1 on autophagy induction with target-specific siRNA. In addition, we also explored changes in the ER stress molecules and UPR pathway associated proteins in 2C- or 3D-overexpressing cells.

## Results

### The recombinant plasmids expressed EMCV proteins

To identify the EMCV proteins involved in autophagy induction, we first amplified the VP1, VP2, VP3, VP4, 2A, 2B, 2C, 3A, 3B, 3C and 3D genes of the EMCV BJC3, GST or LC3 genes by RT-PCR or PCR and constructed corresponding recombinant plasmids with HA tags or GFP tags. The amplified product for each gene was consistent with the size as expected (Additional file 1: Figure S1A). The expression of each EMCV protein by the recombinant plasmids was examined in BHK-21 cells through transfection and Western blotting analysis. The EMCV proteins were expressed effectively except for the 3B gene (Additional file 1: Figure S1B). The transfected BHK-21 cells were simultaneously used to analyze subsequently the expression levels of LC3-I, LC3-II and p62.

### Puncta accumulated in the BHK-21 cells that were co-transfected with the EMCV protein-expressing plasmid and pEGFP-LC3

**Figure 1:**
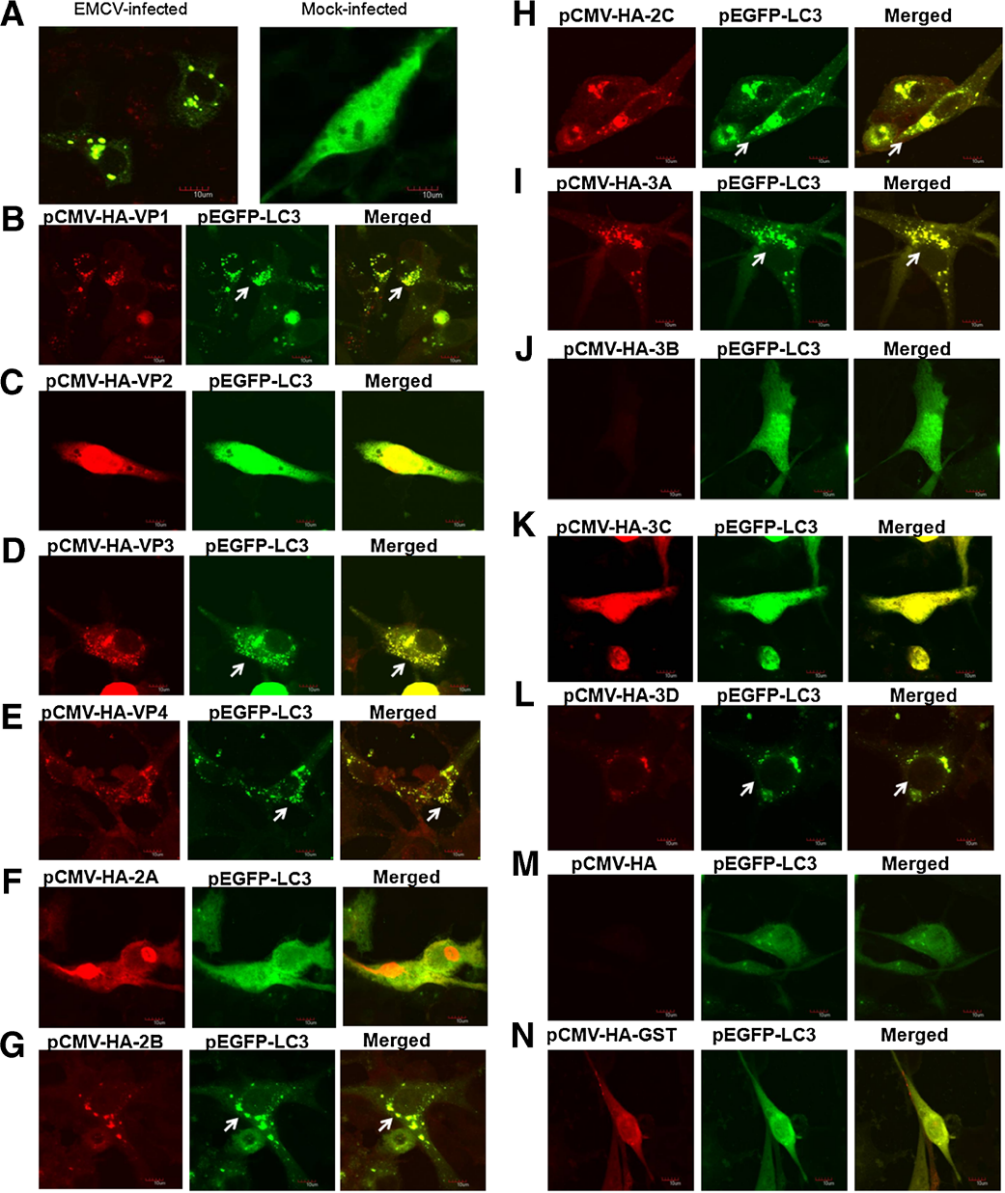
**Immunofluorescence puncta in the cytoplasm of BHK-21 cells that were co-transfected with each HA-tagged EMCV protein-expressing plasmid and pEGFP-LC3. (A)** Confocal immunofluorescence analysis was performed on BHK-21 cells transfected with the pEGFP-LC3 plasmid at 18 h after transfection, and the cells were then infected with the EMCV BJC3 strain for 12 h (MOI = 0.005). A GFP-LC3 signal (green) and EMCV VP1 protein staining (red) are shown. **(B-N)** The BHK 21 cells were co-transfected with each HA-tagged EMCV protein-expressing plasmid or GST protein-expressing plasmid and pEGFP-LC3. At 48 h after transfection, the cells were fixed and processed by immunostaining with a mouse monoclonal antibody against HA and goat anti-mouse secondary antibodies conjugated to TRITC. The HA (red) and GFP-LC3 (green) proteins were examined by immunoconfocal microscopy. The white arrows show the positive puncta of LC3 proteins or the colocalization of the LC3 proteins and EMCV proteins. The scale bars in panels **(A-N)** represent 10 μm.

The LC3 is a specific marker protein for monitoring autophagic vesicle formation through its vesicle formation and lipidation reaction [26]. To facilitate the observation of autophagic vesicles by fluorescence microscopy, BHK-21 cells were transfected with a green fluorescent protein-tagged LC3 plasmid (GFP-LC3). The patterns of the GFP-LC3 in BHK-21 cells were diffuse. By contrast, the green fluorescent pattern exhibited puncta morphology upon EMCV infection, resembling the pattern of autophagosome-like vesicles, which suggests that the BHK-21 cells that were transfected and were expressing GFP-LC3 were suitable for studying autophagy (Figure 1A).

To assess which viral protein could trigger autophagy, plasmids expressing various HA-tagged EMCV proteins were co-tranfected with pEGFP-LC3 into BHK-21 cells. The fluorescence microscopy was performed to provide a puncta analysis of the co-transfected cells. The results showed that the BHK-21 cells that were co-transfected with the VP1-, VP3-, VP4-, 2B-, 2C-, 3A- or 3D-expressing plasmids and pEGFP-LC3 exhibited a number of positive puncta that were distributed in the cytoplasm, and the VP1, VP3, VP4, 2B, 2C, 3A or 3D colocalized with a subset of GFP-LC3 puncta (Figure 1B, 1D, 1E, 1G, 1H, 1I, and 1L), whereas the BHK-21 cells that were co-transfected with the VP2-, 2A- or 3C-expressing plasmids and pEGFP-LC3 and mock-transfected with the pEGFP-LC3 and pCMV-HA displayed no positive puncta (Figure 1C, 1F, 1K, and 1M). In addition, theexpression of 3B was not observed in the BHK-21 cells co-transfected with the 3B--expressing plasmid and pEGFP-LC3 (Figure 1J), which was consistent with the result of Western blotting analysis.

It was necessary to rule out the possibility that positive puncta are elicited by VP1, VP3, VP4, 2B, 2C, 3A and 3D due to the overloaded protein expression. In comparison with the cells expressing VP1, VP3, VP4, 2B, 2C, 3A and 3D, the cells that were expressing glutathione S-transferase (GST) as a irrelevant protein control exhibited no positive puncta (Figure 1N).

### Nonstructural proteins 2C and 3D increased the autophagic activity and the formation of autophagosome-like vesicles

**Figure 2:**
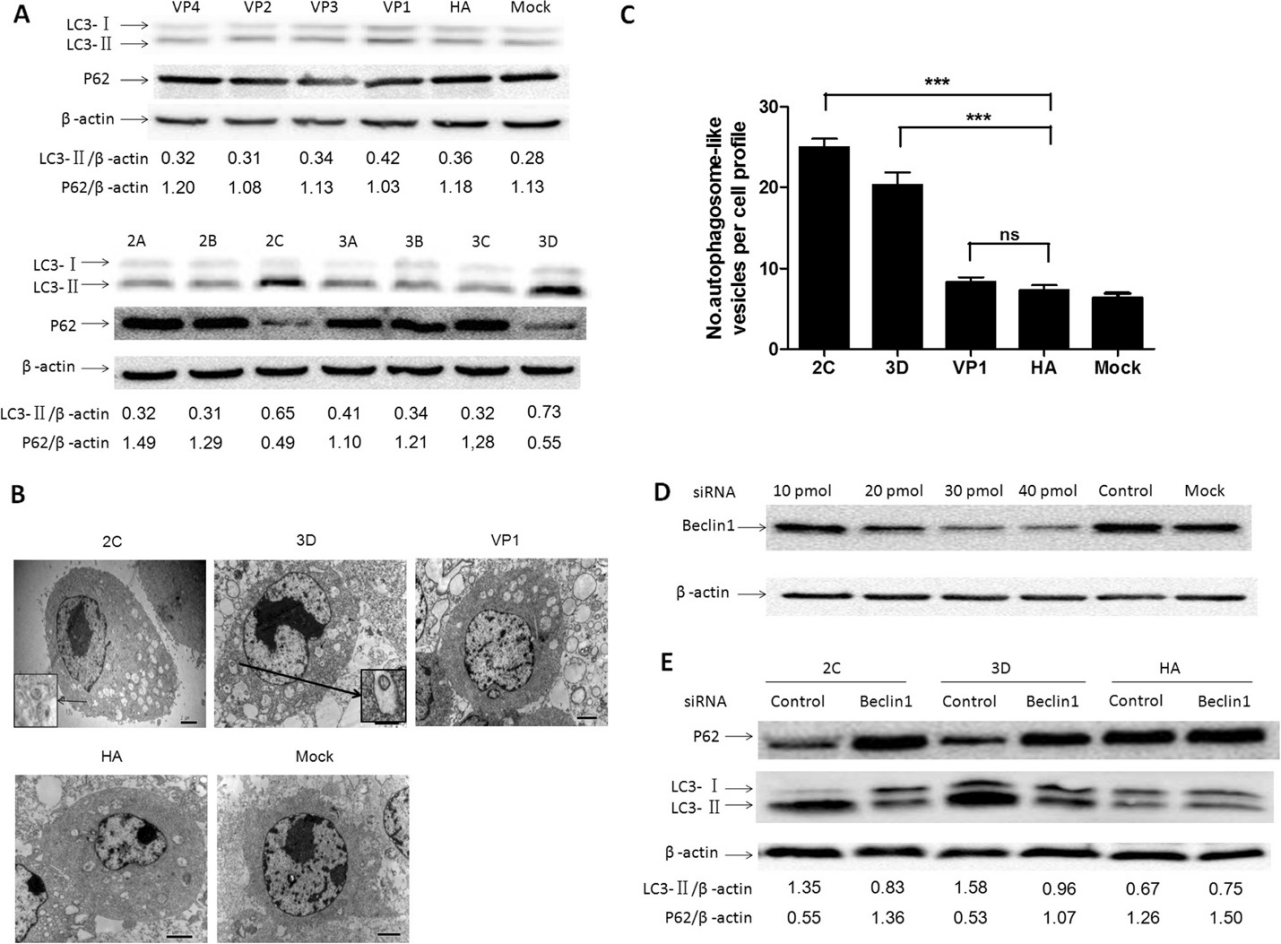
**Nonstructural proteins 2C and 3D increased autophagic activity and the formation of autophagosomes. (A)** Western blotting analyses of LC3, p62 and β-actin in BHK-21 cells that were transfected individually with an HA-tagged EMCV protein-expressing plasmid. HA, the BHK-21 cells transfected with pCMV-HA; Mock, the untransfected BHK-21 cells. The band intensities of LC3-II, p62 and β-actin were quantified, and the relative ratios of LC3-II/β-actin and p62/β-actin are shown in the lower blots. **(B)** The BHK-21 cells were transfected with pCMV-HA-2C, pCMV-HA-3D, pCMV-HA-VP1 or pCMV-HA and mock (untransfected BHK-21 cells) for 48 h, and they were fixed, processed, and imaged by transmission electron microscopy (TEM). The morphologically characteristic double-membrane vesicles are indicated by black arrows in the relevant areas. Magnification, 10,000×; scale bars, 2 μm. **(C)** A quantification of the number of autophagosome-like vesicles per cell profile in BHK-21 cells that were transfected with 2C-, 3D-expressing plasmid, pCMV-HA-VP1 or pCMV-HA and mock (untransfected BHK-21 cells) Data are the means ± SD (error bars) for 8 cells per experimental condition from three independent experiments. ***p < 0.001, compared with the control cells. **(D)** BHK-21 cells were transfected with 10, 20, 30 or 40 pmol Beclin1-siRNA or 40 pmol control-siRNA, and then Beclin1 protein was detected by western blotting at 48 h post-transfection. Mock, the untransfected BHK-21 cells. **(E)** BHK-21 cells transfected with 40 pmol Beclin1-siRNA or control-siRNA. After 48 h, the knockdown cells were reseeded, transfected with pCMV-HA-2C, pCMV-HA-3D or pCMV-HA for an additional 48 h and processed for Western blotting analysis. The band intensities of LC3-II, p62 and β-actin were quantified, and the relative ratios of LC3-II/β-actin and p62/β-actin are shown in the lower blots.

Upon autophagosome formation, LC3 is known to be converted from its cytosolic form (LC3-I) to the lipidated, autophagosome-associated form (LC3-II), which exhibits faster mobility on SDS-polyacrylamide gels. The increased level of LC3-II has been used to reflect the degree of autophagosome formation in each sample [26]. P62 is a link protein between LC3 and ubiquitinated substrates, and it is specifically degraded by autolysosomes, which is considered a marker for autophagic degradation activity [25, 27]. We further analyzed the expression levels of LC3-I, LC3-II and p62 in the BHK-21 cells transfected with the recombinant plasmid expressing each EMCV protein by Western blotting. The results demonstrated that the LC3-II level significantly increased and p62 clearly degraded in the BHK-21 cells transfected with 2C-or 3D-expressing plasmids (Figure 2A). The densitometry ratio of LC3-IIand β-actin in 2C- or 3D-expressing cells was much higher than it was in the other EMCV protein-expressing cells and mock-transfected BHK-21 cells, suggesting that a clear increase in autophagosome formation occurred in the BHK-21 cells that were transfected with a 2C- or 3D-expressing plasmid. The relative p62/β-actin ratios were quantified and significantly decreased in the BHK-21 cells expressing 2C- or 3D, leading to the enhanced autophagic activity.

To determine further whether autophagy is triggered in the BHK-21 cells that were transfected with a 2C- or 3D-expressing plasmid, TEM was performed to provide an ultrastructural analysis of the transfected cells. Previous studies have shown that the VP1 of EMCV can colocalize with LC3 during viral infection [25]. Our present finding showed that GFP-LC3-positive puncta could be simultaneously induced and colocalized with VP1 in the BHK-21 cells that were transfected with pCMV-HA-VP1 (Figure 1B). Thus, in our study, the BHK-21 cells expressing EMCV VP1 that did not induce autophagy were used as a control to analyze the autophagosome-like vesicle formation ability as induced by 2C or 3D. As shown in Figure 2B, the BHK-21 cells that were transfected with the 2C- or 3D-expressing plasmid had significantly increased double- or single-membrane vesicles in the cytoplasm in comparison with the cells that were transfected with the pCMV-HA-VP1 plasmid (as a control of viral protein that did not induce autophagy) or pCMV-HA plasmid (as a control) and mock-transfected cells; meanwhile, the recognizable cytoplasmic contents or degraded organelles seemed to be sequestered in most of the well-defined vesicles with morphologically typical autophagic vacuoles within the cells. Further quantitative analyses indicated that there was a significant increase in the number of autophagosome-like vesicles in the cytoplasm of the cells transfected with 2C- or 3D-expressing plasmid (Figure 2C).

To verify the possibility that LC3 modification was caused by autophagic signalling instead of reflecting an EMCV 2C- or 3D-induced membrane alteration, the knockdown effect of Beclin1 (one of the crucial factors for autophagosome formation) on the induced LC3 and p62 levels was assessed [28]. We used specific siRNA to silence the Beclin1 gene in BHK-21 cells. The cells treated with specifically targeted Beclin1 siRNA showed a dose-dependent reduction in the Beclin1 protein level (Figure 2D), and 40 pmol of siRNA was then used for further study. The increased levels of LC3-II bands and degraded p62 levels in response to 2C or 3D over-expression were significantly inhibited upon Beclin1 silencing when compared with the control groups (Figure 2E), indicating that EMCV 2C and 3D can induce autophagy signalling.

Taken together, we found that EMCV 2C or 3D protein exhibited clear abilities to induce the conversion of LC3, degrade p62 and increase the number of autophagosome-like vesicles in BHK-21 cells in comparison with control cells expressing other proteins. Thus, our further studies of autophagy mechanisms primarily focused on analyzing 2C and 3D proteins.

### ER Stress was induced in EMCV-infected or EMCV 2C- or 3D-expressing BHK 21 cells

**Figure 3:**
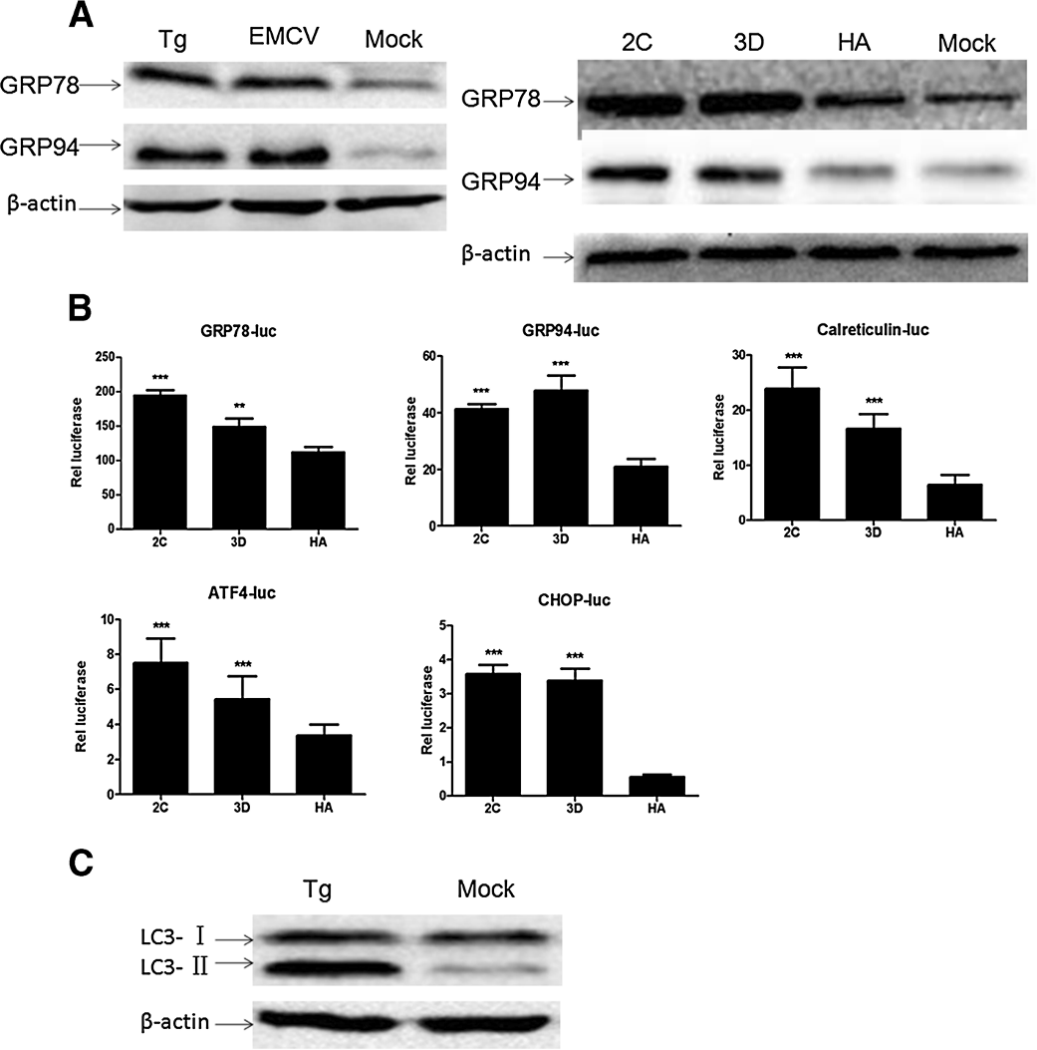
**ER Stress is activated in BHK-21 cells transfected with 2C- or 3D-expressing plasmid. (A)** A Western blotting analysis of GRP78, GRP94 and β-actin in the extracts of BHK-21 cells that were treated with 500 nM Tg for 12 h, infected with EMCV (MOI = 0.005) for 12 h or transfected with 2C- or 3D-expressing plasmid for 48 h. **(B)** The BHK-21 cells were transfected with pGL3-GRP78-luc, pGL3-GRP94-luc, pGL3-Carlreticulin-luc, pGL3-ATF4-luc or pGL3-CHOP-luc along with pRL-TK and pCMV-HA-2C, pCMV-HA-3D or pCMV-HA. The firefly luciferase activity was measured in cell lysates that were normalized with Renilla luciferase activities at 48 h post-transfection. Data are expressed as the means ± SD. **p < 0.01; ***p < 0.001, compared with the corresponding control. HA, the BHK-21 cells transfected with pCMV-HA and Mock, the uninfected or untransfected BHK-21 cells, as the control. **(C)** The conversion of LC3 by ER-stress inducer Tg. BHK-21 cells without treatment or with a 500 nM Tg treatment for 12 h were analyzed by Western blotting for LC3 and β-actin.

Because many picornaviruses can induce ER stress [29, 30] and this ER stress induces autophagy [31–33], EMCV might cause ER stress and therefore induce autophagy. To test this hypothesis, we analyzed whether the marker molecules of the ER stress were regulated in BHK-21 cells by EMCV infection, 2C or 3D protein expression or thapsigargin (Tg) treatment (as a positive control), which is a known ER stress inducer [34]. As expected, when compared with the control cell groups, western blotting analyses showed that EMCV infection, 2C or 3D protein expression or Tg treatment could enhance the levels of ER stress marker proteins GRP78 and GRP94 (Figure 3A). Moreover, we also found that 2C or 3D altered the ER homeostasis, which was reflected in the induction of the ER stress marker, such as DNA damage-inducible gene 153 (GADD153/CHOP), ER chaperones GRP78 and GRP94, calreticulin and the activating transcription factor (ATF4), according to the luciferase assays. As shown in Figure 3B, the relative luciferase activity of these protein promoters were significantly activated in the BHK-21 cells that were transfected with 2C- or 3D-expressing plasmid compared with the transfected cells with control plasmid pCMV-HA, suggesting that EMCV 2C and 3D can trigger the up-regulation of ER stress molecules at the transcriptional level.

To detect whether the activation of ER stress induces autophagy in BHK-21 cells, we analyzed the autophagy level in BHK-21 cells treated with Tg, an ER stress inducer (Figure 3C). The conversion of LC3 was significantly enhanced by Tg in comparison with the mock-treated cells, indicating that ER stress can induce the autophagy signaling of BHK-21 cells. The above results demonstrated that EMCV and 2C or 3D protein could induce ER stress, and the activation of ER stress induced by Tg could enhance the autophagy signaling. Therefore, we inferred that EMCV and 2C or 3D protein might induce autophagy via activating ER stress in BHK-21 cells.

### Regulating the UPR pathway in the EMCV-infected or EMCV 2C- or 3D-expressing BHK 21 cells

**Figure 4:**
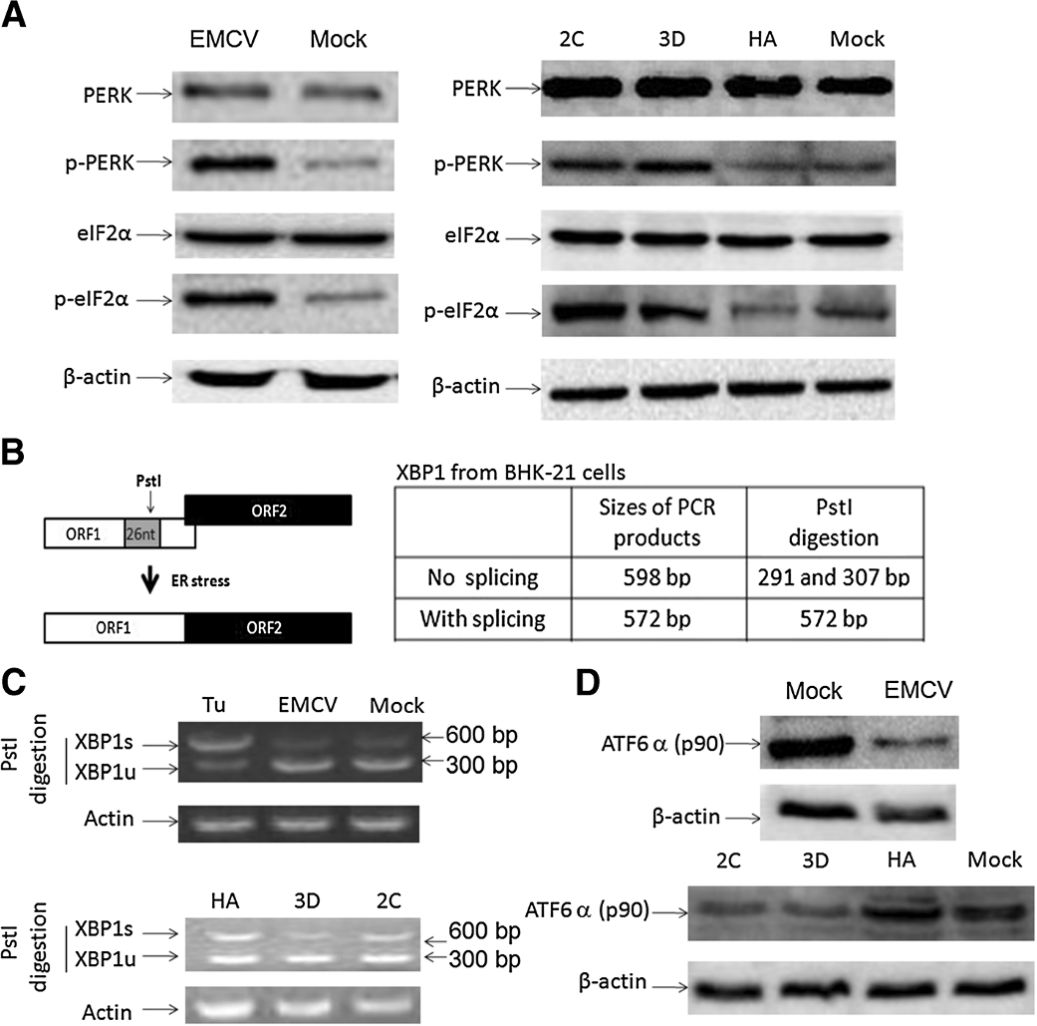
**The regulation of the UPR pathway in the EMCV-infected or with expressed EMCV 2C or 3D BHK 21 cells. (A)** BHK-21 cells were harvested after infecting with EMCV (MOI = 0.005) for 12 h or transfecting with pCMV-HA-2C, pCMV-HA-3D or pCMV-HA (as a control) for 48 h, and subjected to Western blotting analysis for PERK, p-PERK, eIF2α, p-eIF2α and β-actin with the indicated antibodies. **(B)** The analysis scheme for XBP1 mRNA splicing. The relative locations of the 26-nt intron and the PstI restriction site are shown. The sizes of PCR-amplified fragments from spliced XBP1 (XBP1s) and unspliced XBP1 (XBP1u) with or without PstI cleavage are also listed. **(C)** RT-PCR analysis. The BHK-21 cells were treated with Tu (1 μg/ml) for 12 h, infected with EMCV (MOI = 0.005) for 12 h or transfected with the pCMV-HA-2C, pCMV-HA-3D or pCMV-HA (as a control) for 48 h. Total cellular RNAs were prepared, and RT-PCR was performed by using specific primers to determine the XBP1 splicing (XBP1s) level. To detect XBP1 splicing (XBP1s), the PCR products were digested with PstI and electrophoresed. The XBP1u (291/307 bp) and XBP1s (572 bp) bands are indicated. **(D)** A Western blotting analysis of ATF6α and β-actin in the cell extracts of BHK-21 cells that were infected with EMCV (MOI = 0.005) for 12 h or transfected with 2C- or 3D-expressing plasmid for 48 h.

In response to ER stress, the UPR pathway is activated and serves to minimize ER malfunction. To dissect the mechanism of autophagy induction by EMCV infection and expression of EMCV 2C or 3D protein, we examined whether EMCV and 2C or 3D protein can induce the PERK pathway. As shown in Figure 4A, EMCV infection and 2C or 3D protein could activate the PERK pathway, as indicated by the levels of PERK, p-PERK, eIF2α and p-eIF2α protein. The cells infected with EMCV and transfected with 2C- or 3D-expressing plasmid exhibited higher expressions of the p-PERK and p-eIF2α proteins in comparison with the mock-infected cells or the cells that were transfected with pCMV-HA and mock cells; nevertheless, the PERK and eIF2α protein levels remained unchanged.

To assess whether EMCV and 2C or 3D protein also induces the IRE1-XBP1 pathway to trigger UPR, we analyzed XBP1 mRNA splicing in BHK-21 cells that were infected with EMCV or were overexpressing 2C or 3D proteins. The XBP1 cDNA was amplified by RT-PCR and digested by PstI, for which there is a restriction site located within the 26-nt region of XBP1 cDNA as removed by IRE1-mediated splicing, as previously described [30, 35] (Figure 4B). Tunicamycin (Tu), an inducer of XBP1 splicing (XBP1s), served as a positive control [35]. We noticed that Tu-treated cells appeared to express high XBP1s, as evidenced by the resistance of the spliced product to PstI digestion. When compared with BHK-21 cells infected with EMCV and mock-infected cells, no obvious differences were observed in the levels of XBP1, which was subtly spliced, indicating that EMCV infection is not able to significantly activate the IRE1-XBP1 pathway substantially through splicing XBP1. Although 2C or 3D proteins could cause low levels of XBP1s in BHK-21 cells tranfected with pCMV-HA-2C and pCMV-HA-3D, no significant differences were shown between the cells expressing 2C or 3D protein and the control cells (Figure 4C), indicating that the overexpression of 2C or 3D protein cannot induce the activation of the IRE1-XBP1 pathway.

ATF6a is cleaved by trans-membrane proteases and translocated to the nucleus, where it activates the genes responsible for the ER stress response [36, 37]. ATF6a cleavage was analysed in BHK-21 cells to assess whether EMCV infection or the expression of EMCV 2C or 3D protein activate the ATF6a pathway. As shown in Figure 4D, an intact form of ATF6a (90 kDa) was detected in the control cells, and in EMCV-infected or 2C- or 3D-expressing BHK-21 cells, a lower level of its intact form was uncovered by the Western blotting assay.

## Discussion

Viruses have developed complex mechanisms for manipulating normal cellular pathways to facilitate viral replication and to evade host defence mechanisms. Recently, several studies have revealed that cellular autophagy is involved in various pathogenic infections and plays a crucial role in these processes [5, 7, 38]. During the infection process, viruses have been shown to employ the autophagic machinery to replicate and survive [39–41]. Further studies have shown that some viruses can induce autophagy by using their proteins, such as the NSP4 of rotavirus [42], the NS4B of hepatitis C virus [24], nonstructural protein p17 of avian reovirus [43] and matrix protein 2 of influenza A virus [44]. Although our previous studies showed that EMCV infection can induce the autophagy process [28], more detailed evidence concerning the EMCV protein(s) involved in virus-induced autophagy remain unknown.

In the present study, we found that the BHK-21 cells that were co-expressing VP1, VP3, VP4, 2B, 2C, 3A or 3D and GFP-LC3 displayed a number of positive puncta in the cytoplasm and these EMCV proteins colocalized with a subset of GFP-LC3 puncta respectively (Figure 1B, 1D, 1E, 1G, 1H, 1I, and 1L), whereas the cells that were expressing other EMCV proteins (VP2, 2A and 3C), the irrelevant protein (GST) and the cell control transfected with the pCMV-HA plasmid exhibited no positive puncta (Figure 1C, 1F, 1K, 1M and 1N). In principle, the formation of GFP-LC3 puncta accumulations represents a component of the autophagy process. However, the formation of ubiquitinated GFP-LC3 positive protein accumulations may be triggered and does not completely imply either the induction of autophagy (or autophagosome formation), or autophagic flux through the system [27]. Therefore, to rule out this possibility, we also detected the LC3 modification and autophagic vesicles by Western blotting analysis and transmission electron microscopy to analyze the activation of the autophagy process (Figure 2A-C). Western blotting analysis showed that the increased level of LC3-II, the reduced expression of p62, the enhanced ratio of LC3-IIto β-actin and the degradated ratio of p62 to β-actin represented a significant increase in the autophagy level of 2C- or 3D-overexpressed cells, whereas no similar results were shown in other viral proteins. The number of autophagosome-like vesicles with various sizes significantly increased in 2C- or 3D-overexpressing cells in comparison with the control cells by transmission electron microscopy. The size difference in autophagosome-like vesicles most likely implied that these vesicles contained different cytoplasmic contents, such as obsolete organelles and cytoplasmic proteins.

To verify that the LC3 modification phenomenon was caused by autophagic signalling instead of 2C- or 3D- induced membrane alterations, we employed a specific siRNA targeting the autophagy-critical gene required for autophagosome formation. Disrupting the class III phosphatidyl inositol 3-kinase (PI3K) signalling complex that was required for autophagosome formation by Beclin1 siRNA clearly reduced the induction level of LC3-II and the degradation of p62 in 2C- or 3D-transfected cells (Figure 2D-E), further demonstrating that EMCV 2C or 3D induced autophagy through the variation in autophagic vesicle formation. A previous study indicated that the 2BC and 3A proteins of poliovirus are responsible for inducing autophagy [45]. Thus, we deduced that these differences in induction ability are most likely explained by the individual expression or overexpression level of various viral proteins in cells or the significant differences between nonstructural proteins encoded by different picornaviruses [46]. Based on our findings, we primarily engaged in further research of autophagy mechanisms to focus on EMCV 2C and 3D proteins.

The endoplasmic reticulum (ER) system, which is a major site for the synthesis and control of the membrane or secreted protein quality, is a primary compartment of signal initiation and transduction for responding to a variety of stimuli, including viral infections [35, 47]. Although studies showed that many picornaviruses induce autophagy by activating ER stress [29, 30], a question remains as to whether the underlying mechanisms of EMCV and 2C or 3D protein-induced autophagy are related to ER stress. Thus, the activation of the ER stress pathway was analyzed in BHK-21 cells infected with EMCV, expressed 2C or 3D protein or treated with Tg (as a positive control) (Figure 3). The results showed that the marker molecules of the ER stress pathway were activated in these BHK-21 cells, indicating that EMCV 2C and 3D proteins are potent ER stress inducers in BHK-21 cells and play an important role in EMCV-activated ER stress. The two proteins could trigger the activation of ER stress marker molecules not only via up-regulation at the transcriptional level but also by activation at the translational level. Interestingly, we found that, ER stress was activated and accompanied by the up-regulation of the autophagy level through an enhanced conversion of LC3 in BHK-21 cells treated with Tg, and we therefore deduced that EMCV 2C or 3D protein also induce autophagy by activating the ER stress.

A number of viruses have been shown to induce ER stress during viral infection. Cells respond to ER stress by activation of the UPR pathway to maintain homeostasis of the ER. However, the pattern of molecular interactions that occurs within the UPR pathway differs. This finding depends on the viral identity and type of host cell. Many viruses clearly induce all the UPR pathways, and some viral infections activate the partial pathway [29, 30, 48]. A detailed analysis of the UPR pathway was undertaken in our study to understand this case (Figure 4). Our results showed that EMCV infection and 2C- or 3D-protein expression could phosphorylate the associated molecules of the PERK pathway and cleave the intact form of ATF6a (90 kDa), but they could not splice XBP1 mRNA, indicating that the activation of the PERK pathway and the ATF6a pathway can transiently block mostly protein translation and regulate the transcription of the ER chaperon against ER malfunction. These pathways are specifically induced to trigger autophagy initiation [49–51]. Moreover, many genes were regulated by activation of the UPR pathway at the transcriptional and translational levels and were involved in recovering from ER stress [52], which may be available for EMCV replication. Although the exact contributions of the host and virus to UPR induction remain unclear, we showed that EMCV 2C or 3D protein induced autophagy by initiating the PERK and ATF6a pathways in response to ER stress, which finally benefits this cellular event and viral replication.

Although the molecular mechanisms regulating autophagic signal pathways to facilitate viral survival and replication by viral proteins are required to be further explored, a growing number of studies have demonstrated that the interactions between viral proteins and the cellular proteins associated with autophagy play important roles in regulating autophagy process. Foot and mouth disease virus (FMDV) nonstructural protein 2C binds to Beclin1, a central regulator of the autophagy pathway, to prevent the fusion of lysosomes to autophagosomes, thereby allowing for viral survival [23]. Additionally, a study showed that the HCV NS4B protein can induce autophagy and promote viral replication through recruiting the Rab5 and Vps34 complex [24]. Thus, analyzing the interaction between viral proteins and autophagy-associated proteins of host cells is worthy for better evaluating the role of autophagy in relation to EMCV infection.

## Conclusions

Taken as a whole, our findings are the first to indicate that nonstructural proteins 2C and 3D are involved in autophagy as induced by EMCV. The fact that Beclin1 knockdown inhibited the conversion of LC3 and degradation of p62 further demonstrated 2C or 3D-inducing functions. Additionally, the 2C- or 3D-induced autophagy was shown to be associated with endoplasmic reticulum (ER) stress pathway via regulating the UPR protein expression. Our present studies help to better understand the EMCV-induced autophagy process.

## Materials and methods

### Cells, virus and plasmids

BHK-21 cells were originally obtained from the American Type Culture Collection (ATCC) and maintained in Dulbecco’s modified Eagle medium (DMEM) supplemented with 10% heat-inactivated foetal bovine serum (FBS), 100 U/ml of penicillin G, and 100 g/ml streptomycin at 37°C in a humidified 5% CO_2_ incubator. An EMCV strain (BJC3) that was isolated in our laboratory was used in this study [53]. Plasmids pEGFP-C1 and pCMV-HA were purchased from Clontech (Mountain View, CA, USA), and plasmids pGL3-Basic and pRL-TK were purchased from Promega (Madison, WI, USA).

### Antibodies and reagents

The following antibodies were used: anti-GRP78, anti-GRP94, anti-ATF6, anti-PERK, anti-phospho-PERK, anti-eIF2α, and rabbit anti-phospho-eIF2α antibody (Cell Signal Technology, Inc., Danvers, MA, USA). The rabbit antibodies against LC3 and p62 and a mouse monoclonal antibody against HA and β-actin, the secondary antibodies tetramethyl rhodamine isothiocyanate TRITC-conjugated goat anti-mouse and horseradish peroxidase conjugated goat anti-mouse or anti-rabbit were purchased from Sigma-Aldrich (St. Louis, MO, USA). Anti-Beclin1 rabbit antibody was obtained from Santa Cruz Biotechnology (Santa Cruz, CA, USA). Mouse anti-VP1 mAb was prepared in our laboratory. Tunicamycin (Tu) and thapsigargin (Tg) were purchased from Sigma-Aldrich. RIPA lysis buffer was obtained from Beyotime (Jiangsu, China). Lipofectamine LTX and PLUS and RNAiMAX reagent were purchased from Invitrogen (Auckland, NY, USA).

### RNA preparation and RT-PCR analysis

Total RNA from cultured cells was isolated with an RNeasy Mini Kit (Qiagen, Hilden, Germany) by following the manufacturer’s protocol. First-strand cDNAs were reverse-transcribed (RT) with 2 μg of RNA as the template, and the target genes were PCR-amplified by following the procedures described in the one-step RT-PCR kit (Qiagen) according to the manufacturer’s recommendations.

The XBP1 and β-actin genes were amplified by RT-PCR with the specific primers listed in Additional file 1: Table S1, and the primers were designed according to the XBP1 and Actin sequences available in the GenBank database (accession no. NM_001244047 and no. XM_006176094, respectively).

### Constructing the expression plasmids for LC3, GST and EMCV genes

The open reading frame fragment of the Atg8 homolog known as the microtubule-associated protein light chain 3 (LC3) gene from BHK-21 cells was amplified by RT-PCR, and the specific primers were designed in accordance with the Atg8 gene sequence in the GenBank database (accession no. XM_003495104) (Additional file 1: Table S1). The amplified cDNA was then subcloned into pEGFP-C1. Each gene of the EMCV protein was amplified by RT-PCR with the primers listed in Additional file 1: Table S1 and cloned into pCMV-HA to generate the following expression plasmids: pCMV-HA-VP1, pCMV-HA-VP2, pCMV-HA-VP3, pCMV-HA-VP4, pCMV-HA-2A, pCMV-HA-2B, pCMV-HA-2C, pCMV-HA-3A, pCMV-HA-3B, pCMV-HA-3C and pCMV-HA-3D. The glutathione S-transferase (GST) gene was amplified by PCR from cloning vector pGEX-6P-1 (accession no. U78872) with the specific primers listed in Additional file 1: Table S1 and then cloned into pCMV-HA to generate recombinant plasmid pCMV-HA-GST. The pGL3-GRP78-luc, pGL3-GRP94-luc, pGL3-calreticulin-luc, pGL3-ATF4-luc, pGL3-CHOP-luc-carrying mouse GRP78, GRP94, calreticulin, ATF4 and CHOP promoters were cloned into the pGL3-basic vector [54]. The plasmids were sequenced to confirm that each amplified product had no errors introduced as a result of PCR amplification.

### Viral infection

In accordance with the requirements of different experiments, the BHK-21 cells were infected with either EMCV BJC3, or they were mock-infected with phosphate-buffered saline (PBS). Following a 1 h absorption period, unattached viruses were removed by aspiration. The cells were then washed thrice with PBS and cultured in complete medium at 37°C for the indicated time points until different samples had been harvested for further experiments.

### Western blotting

A whole cell lysate of BHK-21 cells was prepared at the indicated time points after transfecting with the RIPA lysis buffer according to the manufacturer’s protocol. The supernatant was stored at −80°C for western blotting. Twenty micrograms of each extract was subjected to electrophoresis on an SDS-polyacrylamide gel and transferred onto an Immobilon-P^SQ^ transfer membrane (Millipore, Billerica, MA, USA). The membrane was probed with the indicated primary antibodies and appropriate secondary antibodies, which were detected by using a chemiluminescence detection kit (Thermo Scientific, Inc., Waltham, MA, USA) and then exposed to a chemiluminescence apparatus (Proteinsimple, Santa Clara, CA, USA).

### Luciferase assays

BHK-21 cells were grown to 70-80% confluence in 24-well culture plates (Corning Inc., NY) and transfected with firefly luciferase reporter vectors along with the internal control Renilla luciferase reporter construct, pRL-TK (firefly luciferase reporter construct and pRL-TK in a ratio of 20:1 for BHK-21 cells) and the indicated EMCV protein recombinant plasmids. Forty-eight hours after the transfection, the cells were harvested and assayed for luciferase activity by using the Dual-Luciferase assay system according to the manufacturer^’^s instructions from Promega (E1910).

### Confocal microscopy

BHK-21 cells that had grown to approximately 70-80% confluence in 24-well culture plates were co-transfected with each EMCV protein-expressing vector, GST protein-expressing plasmid (as a control) and pEGFP-LC3 for 48 h, or they were infected with EMCV after transfecting with pEGFP-LC3 plasmid for 12 h. The cells were fixed with pre-cooled 4% paraformaldehyde at the indicated times. The cells were then washed three times with PBS (pH 7.4), and a mouse monoclonal antibody against HA was allowed to incubate with the cells for 2 h at 37°C. The cells were washed three times with PBS and then incubated for 2 h at 37°C with a 1:200 dilution of goat anti-mouse secondary antibodies conjugated to TRITC. Finally, the cells were washed with PBS and directly visualized under a Nikon TE-2000E confocal immunofluorescence microscope (Nikon Instruments, Inc., Melville, NY, USA).

### Transmission electron microscopy (TEM)

Sub-confluent monolayers of BHK-21 cells grown on 6-well culture plates (Corning Inc.) were transfected with pCMV-HA-2C, pCMV-HA-3D, pCMV-HA-VP1 or pCMV-HA and mock-transfected cells for 48 h. The cell samples were then processed as previously described [39]. Ultrathin sections were prepared, examined and imaged under a Hitachi H-7500 transmission electron microscope (Hitachi Ltd; Japan).

### RNA interference (RNAi) knockdown of Beclin1

siRNAs designed by the GenePharma Company (Shanghai, China) were used to knock down Beclin1 in BHK-21 cells. The siRNA sequences included siBeclin1 (sense, 5′-CGGGAAUACAGUGAAUUUATT-3′; antisense, 5′-UAAAUUCACUGUAUUCCCGTT-3′) and negative control siRNA (as a control) (sense, 5′-UUCUCCGAACGUGUCACGUTT-3′; antisense, 5′-ACGUGACACGUUCGGAGAATT-3′). The BHK-21 cells were dissociated and mixed with different concentrations of siRNA by using Lipofectamine RNAiMAX reagent as recommended by the manufacturer’s protocol. The cells were harvested for further analysis after 48 h.

### Statistical analysis

The data were expressed as means ± standard deviations or standard errors as indicated. All statistical analyses were performed by Student’s t-test, and a *p* < 0.05 was considered to be statistically significant

## Electronic supplementary material

Additional file 1: **Supplementary data. Figure S1.** The amplification of EMCV genes and expression analyses by using recombinant plasmids. **(A)** The amplification of the EMCV gene, LC3 and GST (fragment) by RT-PCR or PCR. **(B)** Western blotting analysis of HA-tagged EMCV proteins as expressed by the recombinant plasmids in transfected BHK-21 cells. HA, the BHK-21 cells transfected with pCMV-HA as a control; mock, the untransfected BHK-21 cells were used as a control. The molecular mass markers (kDa) are shown on the left. The size for each EMCV protein with HA-tag was 32 kDa (VP1), 31 kDa (VP2), 26 kDa (VP3), 10 kDa (VP4), 19 kDa (2A), 17k Da (2B), 39 kDa (2C), 12 kDa (3A), 4 kDa (3B), 24 kDa (3C) and 54 kDa (3D). **Table S1.** Primers used for amplifying and sequencing the EMCV genes and the autophagy pathway-associated genes and gene promoters. (DOCX 1 MB)
