# Supplementary material for: Nonstructural proteins 2C and 3D are involved in autophagy as induced by the encephalomyocarditis virus
Source: Virol J. 2014 Sep 1;11:156. doi: 10.1186/1743-422X-11-156 (PMC4161894; doi:10.1186/1743-422X-11-156)
Supplement: Supplementary file 1 — Additional file 1: Supplementary data. Figure S1. The amplification of EMCV genes and expression analyses by using recombinant plasmids. (A) The amplification of the EMCV gene, LC3 and GST (fragment) by RT-PCR or PCR. (B) Western blotting analysis of HA-tagged EMCV proteins as expressed by the recombinant plasmids in transfected BHK-21 cells. HA, the BHK-21 cells transfected with pCMV-HA as a control; mock, the untransfected BHK-21 cells were used as a control. The molecular mass markers (kDa) are shown on the left. The size for each EMCV protein with HA-tag was 32 kDa (VP1), 31 kDa (VP2), 26 kDa (VP3), 10 kDa (VP4), 19 kDa (2A), 17k Da (2B), 39 kDa (2C), 12 kDa (3A), 4 kDa (3B), 24 kDa (3C) and 54 kDa (3D). Table S1. Primers used for amplifying and sequencing the EMCV genes and the autophagy pathway-associated genes and gene promoters. (DOCX 1 MB) [file 12985_2014_2482_MOESM1_ESM.docx]

**
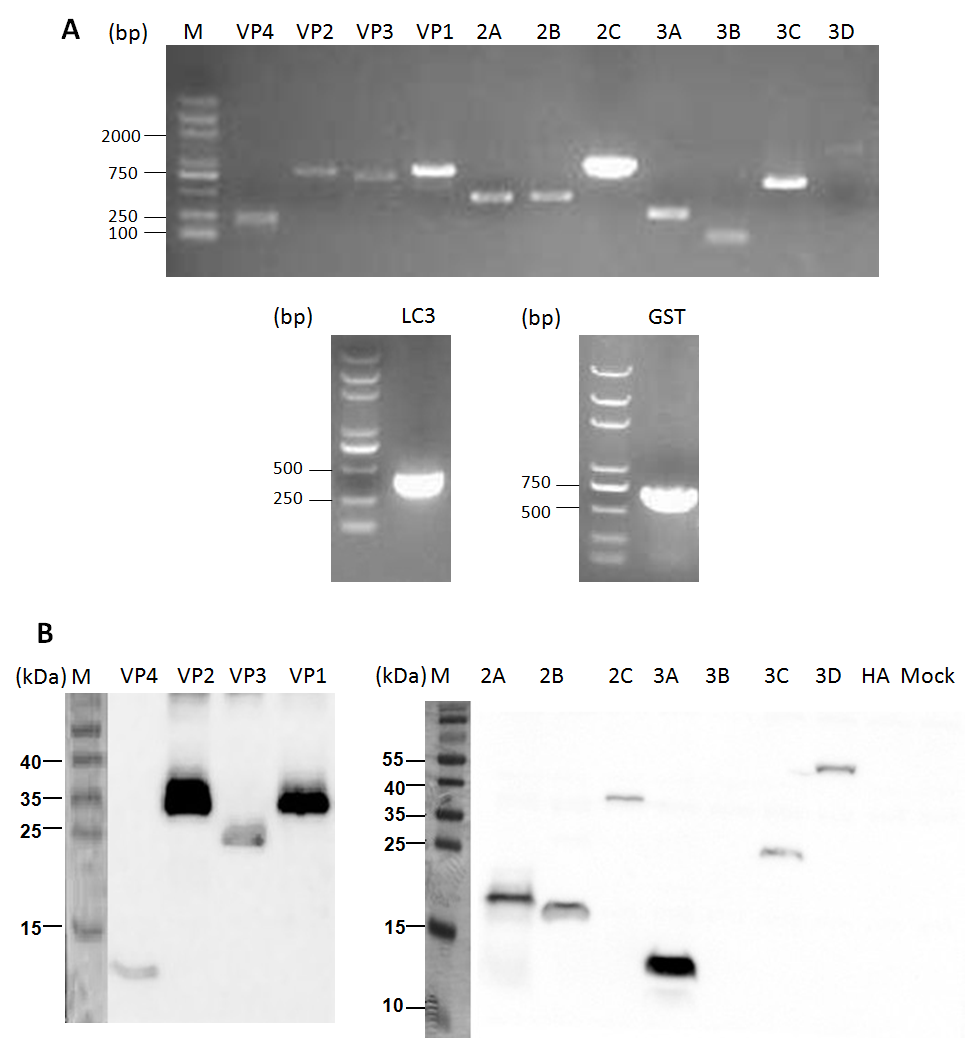
Supplementary data**

**Fig. S1. The amplification of EMCV genes and expression analyses by using recombinant plasmids.** (A) The amplification of the EMCV gene, LC3 and GST (fragment) by RT-PCR or PCR. (B) Western blotting analysis of HA-tagged EMCV proteins as expressed by the recombinant plasmids in transfected BHK-21 cells. HA, the BHK-21 cells transfected with pCMV-HA as a control; mock, the untransfected BHK-21 cells were used as a control. The molecular mass markers (kDa) are shown on the left. The size for each EMCV protein with HA-tag was 32 kDa (VP1), 31 kDa (VP2), 26 kDa (VP3), 10 kDa (VP4), 19 kDa (2A), 17k Da (2B), 39 kDa (2C), 12 kDa (3A), 4 kDa (3B), 24 kDa (3C) and 54 kDa (3D).

**Table S1** Primers used for amplifying and sequencing the EMCV genes and the autophagy pathway-associated genes and gene promoters

| Primer^a^ | Sequence (5′-3′)^b^ | Size (bp) |
| --- | --- | --- |
| VP1F | TAGAATTCCC*ATG*GGCAATTCCACCTCCTCAG (*Eco*RⅠ) | 831 |
| VP1R | AGGGTACCTCAAGCTAGCAATGGAAGCAT (*Kpn*Ⅰ) |  |
| VP2F | GCGAATTCCC*ATG*GATCAAAATACAGAAGAA (*Eco*RⅠ) | 768 |
| VP2R | TGAGATCTATCACTGTCTAGAAAGTGTCTCGT (*Bgl* Ⅱ) |  |
| VP3F | CTGAATTCTT*ATG*TCGCCCATTCCGGTCAC (*Eco*RⅠ) | *693* |
| VP3R | TAAGATCTATCACTGAGGGCTCCAGGGG (*Bgl* Ⅱ) |  |
| VP4F | CTGAATTCGG*ATG*GGAGTAGAAAACGCTGAA (*Eco*RⅠ) | 210 |
| VP4R | ATGGTACCTCACTCTAGCATCAAGACTCCA (*Kpn*Ⅰ) |  |
| 2AF | CGGAATTCGG*ATG*AGTCCAAATGCCCTAGACAT (*Eco*RⅠ) | 429 |
| 2AR | ATGGTACCTCACCCTGGATTTGTCTCAATG (*Kpn*Ⅰ) |  |
| 2BF | CTGAATTCGG*ATG*CCCTTCATGTTTAGACC (*Eco*RⅠ) | 450 |
| 2BR | GCGGTACCTCATTGTTGTTGGAAAAGAG (*Kpn*Ⅰ) |  |
| 2CF | ATGAATTCGG*ATG*TCCCCCTTGAAACAGGT (*Eco*RⅠ) | 975 |
| 2CR | TCAGATCTATCATTGTGCCACAAGGGTCTGC (*Bgl* Ⅱ) |  |
| 3AF | CTGAATTCTT*ATG*GCTCCAGTAGACGAGGT (*Eco*RⅠ) | 264 |
| 3AR | GCAGATCTATCACTGCTCCTGCTCATCCAAT (*Bgl* Ⅱ) |  |
| 3BF | GTGAATTCGG*ATG*GGACCTTACAATGAGAC (*Eco*RⅠ) | 60 |
| 3BR | TAGGTACCTCACTGAATGTCCAACAACT (*Kpn*Ⅰ) |  |
| 3CF | ATGAATTCGG*ATG*GGACCAAACCCTGTGAT (*Eco*RⅠ) | 615 |
| 3CR | ATTGGTACCTCACTGTGGCTCAAAGGCAT (*Kpn*Ⅰ) |  |
| 3DF | GTGAATTCTT*ATG*GGTGCTCTCGAGAGATT (*Eco*RⅠ) | 1383 |
| 3DR | TAAGATCTATCACTACCAGAACAGACTCC (*Bgl* Ⅱ) |  |
| LC3F | CTGAATTCT*ATG*CCGTCCGAGAAGACCT (*Eco*RⅠ) | 378 |
| LC3R | ATGTCGACTTACACAGCCATTGCTGTC (*Sal*Ⅰ) |  |
| Grp78-promoterF | TATGAGCTCTGACACGCAGACCCCACTCCAGT (*Sac*Ⅰ) | 600 |
| GRP78-promoterR | TATAAGCTTGCCGGCGCTGAGGACCAGTCGCT (*Hin*d Ⅲ) |  |
| GRP94-promoterF | ATTGAGCTCGACCCGCGTCGTAGACGAGAAAAG (*Sac*Ⅰ) | 550 |
| GRP94-promoterR | TATAAGCTTGGTGCGTGGCCGGCGAGT (*Hin*d Ⅲ) |  |
| Calreticulin-promoterF | TTGAGCTCTGCAGGACAGACGGAACGTGAAAG (SacⅠ) | 608 |
| Calreticulin-promoterR | ATAAGCTTCGGCGGCACACAGGCCTTTTAAGAC (*Hin*d Ⅲ) |  |
| ATF4-promoterF | GCGCTAGCGAATTCAGGATAGCAGTATTCTTCTAAG (*Nhe*Ⅰ) | 2500 |
| ATF4-promoterR | ATAAGCTTAAAGCTCTGACGGTCAAAGCCGAAGCTG (*Hin*d Ⅲ) |  |
| CHOP-promoterF | ATAGAGCTCGGGAGGTGGGCAGACAAGTT (*Sac*Ⅰ) | 585 |
| CHOP-promoterR | ACAAGCTTCAGGTTCCGGCTGTTATTCTGGCT (*Hin*d Ⅲ) |  |
| XBP1F | AAACAGAGTAGCAGCGCAGACTGC | 598 |
| XBP1R | GGATCTCTAAGACTAGAGGCTTGGTG |  |
| ActinF | tcctgtggcatccacgaaact | 315 |
| ActinR | gaagcatttgcggtggacgat |  |
| GSTF | GCGAGATCTGGATGTCCCCTATACTAGG(*Bgl* Ⅱ) | 735 |
| GSTR | TATGGTACCTCAGTCACGATGCGGCCGCT(*Kpn*Ⅰ) |  |

^a^ F denotes a forward PCR primer; R denotes a reverse PCR primer. ^b^ The restriction sites are underlined and specified in parentheses at the end of the sequence. The sequence of the eukaryotic transcriptional promoter is shown in italics.
